# Bis(2-{[2-(isopropyl­aza­nium­yl)eth­yl]imino­meth­yl}-6-meth­oxy­phenolato)copper(II) bis­(thio­cyanate)

**DOI:** 10.1107/S1600536811001322

**Published:** 2011-01-15

**Authors:** Lin Yuan

**Affiliations:** a College of Chemistry and Biology Engineering, Yichun University, Yichun 336000, People’s Republic of China

## Abstract

The asymmetric unit of the title compound, [Cu(C_13_H_20_N_2_O_2_)_2_](NCS)_2_, contains one half-dication, located on an inversion center, and one thio­cyanate anion. Each Cu^II^ atom is four-coordinated by two phenolate O and two imine N atoms from two symmetry-related Schiff base 2-{[2-(isopropyl­aza­nium­yl)eth­yl]imino­meth­yl}-6-meth­oxy­phenolate (*L*) ligands in a distorted square-planar geometry. The ammonium groups are involved in the formation of N—H⋯O and N—H⋯N hydrogen bonds, which link one dication and two anions into an electroneutral cluster. When very weak Cu—N interactions with a distance of 2.910 (5) Å between the metal and the thiocyanate anions in apical positions are considered, the secondary coordination polyhedron is a very elongated CuN_4_O_2_ octahedron.

## Related literature

For background to copper(II) complexes with Schiff base ligands, see: Fernandez *et al.* (2010 ▶); Biswas *et al.* (2010 ▶); Chakraborty *et al.* (2010 ▶). For related complexes, see: Ji & Lu (2010 ▶); Cai (2009 ▶); Xia *et al.* (2008 ▶); Suleiman Gwaram *et al.* (2010 ▶); Ma (2008 ▶).
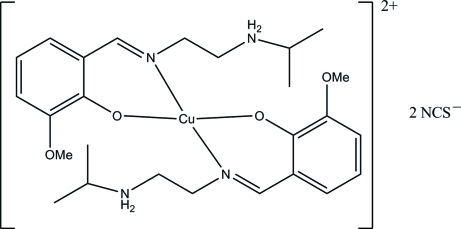


## Experimental

### 

#### Crystal data

[Cu(C_13_H_20_N_2_O_2_)_2_](NCS)_2_
*M* *_r_* = 652.32
Orthorhombic, 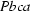

*a* = 13.5307 (12) Å
*b* = 9.7992 (9) Å
*c* = 24.114 (2) Å
*V* = 3197.3 (5) Å^3^
*Z* = 4
Mo *K*α radiation
μ = 0.86 mm^−1^
*T* = 298 K
0.20 × 0.18 × 0.15 mm

#### Data collection

Bruker SMART CCD area-detector diffractometer
Absorption correction: multi-scan (*SADABS*; Sheldrick, 1996 ▶) *T*_min_ = 0.848, *T*_max_ = 0.882
13234 measured reflections
2406 independent reflections
1666 reflections with *I* > 2σ(*I*)
*R*_int_ = 0.063
θ_max_ = 23.8°

#### Refinement

*R*[*F* ^2^ > 2σ(*F* ^2^)] = 0.078
*wR*(*F* ^2^) = 0.143
*S* = 1.15
2406 reflections
190 parameters
6 restraints
H-atom parameters constrained
Δρ_max_ = 0.38 e Å^−3^
Δρ_min_ = −0.36 e Å^−3^

### 

Data collection: *SMART* (Bruker, 1998 ▶); cell refinement: *SAINT* (Bruker, 1998 ▶); data reduction: *SAINT*; program(s) used to solve structure: *SHELXS97* (Sheldrick, 2008 ▶); program(s) used to refine structure: *SHELXL97* (Sheldrick, 2008 ▶); molecular graphics: *SHELXTL* (Sheldrick, 2008 ▶); software used to prepare material for publication: *SHELXL97*.

## Supplementary Material

Crystal structure: contains datablocks global, I. DOI: 10.1107/S1600536811001322/cv5037sup1.cif

Structure factors: contains datablocks I. DOI: 10.1107/S1600536811001322/cv5037Isup2.hkl

Additional supplementary materials:  crystallographic information; 3D view; checkCIF report

## Figures and Tables

**Table 1:** Hydrogen-bond geometry (Å, °)

*D*—H⋯*A*	*D*—H	H⋯*A*	*D*⋯*A*	*D*—H⋯*A*
N2—H2*A*⋯N3	0.90	2.13	2.972 (10)	155
N2—H2*B*⋯O1^i^	0.90	1.87	2.665 (6)	147

Symmetry code: (i) 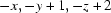.

**Fig. 1.**
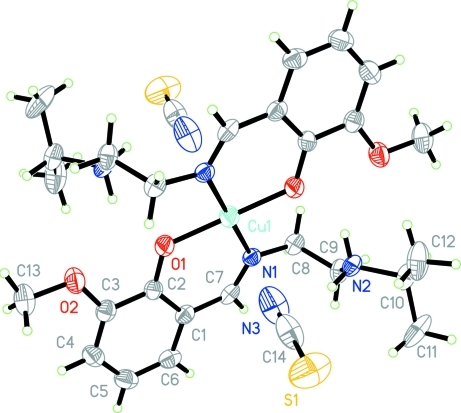
Molecular structure of (I) showing the atomic numbering and 30% probability displacement ellipsoids. Unlabelled atoms are related with labelled ones by symmetry operation (- x, 1 - y, 2 - z).

## supplementary crystallographic information

### Comment

Copper(II) complexes with Schiff bases have been extensively studied (Fernandez *et al.*, 2010; Biswas *et al.*, 2010; Chakraborty *et al.*, 2010). In this paper, we present the title compound (I) - a new copper(II) complex with the Schiff base ligand 2-[(2-isopropylaminoethylimino)methyl]-6-methoxyphenol.

In (I) (Fig. 1), the Cu center is four-coordinated by two phenolate O and two imine N atoms from two Schiff base ligands in a distorted square-planar geometry. The coordinate bond lengths and angles are comparable with those observed in similar complexes (Ji & Lu, 2010; Cai, 2009; Xia *et al.*, 2008; Suleiman Gwaram *et al.*, 2010; Ma, 2008). Ammonium groups are involved in formation of N—H···O and N—H···N hydrogen bonds (Table 1), which link one dication and two anions into electroneutral cluster, where thiocyanate anions can also be considered as ligands coordinating Cu center in apical positions [Cu1—N3 2.910 (5) Å].

### Experimental

Equimolar quantities (0.1 mmol each) of 3-methoxysalicylaldehyde, *N*-isopropylethane-1,2-diamine, ammonium thiocyanate, and Cu(CH_3_COO)_2_.H_2_O were mixed and stirred in methanol for 30 min at reflux. After keeping the filtrate in an air for a few days, blue block crystals were formed.

### Refinement

H atoms were placed in calculated positions and constrained to ride on their parent atoms, with C—H distances in the range 0.93–0.97 Å, N—H distances of 0.90 Å, and with *U*_iso_(H) = 1.2*U*_eq_(C,*N*) and 1.5*U*_eq_(methyl C).

### Crystal data

**Table:**

[Cu(C_13_H_20_N_2_O_2_)_2_](NCS)_2_	*D*_x_ = 1.355 Mg m^−^^3^
*M**_r_* = 652.32	Mo *K*α radiation, λ = 0.71073 Å
Orthorhombic, *P**b**c**a*	Cell parameters from 1594 reflections
*a* = 13.5307 (12) Å	θ = 2.3–24.5°
*b* = 9.7992 (9) Å	µ = 0.86 mm^−^^1^
*c* = 24.114 (2) Å	*T* = 298 K
*V* = 3197.3 (5) Å^3^	Block, blue
*Z* = 4	0.20 × 0.18 × 0.15 mm
*F*(000) = 1372	

### Data collection

**Table:**

Bruker SMART CCD area-detector diffractometer	2406 independent reflections
Radiation source: fine-focus sealed tube	1666 reflections with *I* > 2σ(*I*)
graphite	*R*_int_ = 0.063
ω scans	θ_max_ = 23.8°, θ_min_ = 2.3°
Absorption correction: multi-scan (*SADABS*; Sheldrick, 1996)	*h* = −15→15
*T*_min_ = 0.848, *T*_max_ = 0.882	*k* = −11→8
13234 measured reflections	*l* = −27→27

### Refinement

**Table:**

Refinement on *F*^2^	Primary atom site location: structure-invariant direct methods
Least-squares matrix: full	Secondary atom site location: difference Fourier map
*R*[*F*^2^ > 2σ(*F*^2^)] = 0.078	Hydrogen site location: inferred from neighbouring sites
*wR*(*F*^2^) = 0.143	H-atom parameters constrained
*S* = 1.15	*w* = 1/[σ^2^(*F*_o_^2^) + 13.3941*P*] where *P* = (*F*_o_^2^ + 2*F*_c_^2^)/3
2406 reflections	(Δ/σ)_max_ < 0.001
190 parameters	Δρ_max_ = 0.38 e Å^−^^3^
6 restraints	Δρ_min_ = −0.36 e Å^−^^3^

### Special details

**Table:**

Geometry. All e.s.d.'s (except the e.s.d. in the dihedral angle between two l.s. planes) are estimated using the full covariance matrix. The cell e.s.d.'s are taken into account individually in the estimation of e.s.d.'s in distances, angles and torsion angles; correlations between e.s.d.'s in cell parameters are only used when they are defined by crystal symmetry. An approximate (isotropic) treatment of cell e.s.d.'s is used for estimating e.s.d.'s involving l.s. planes.
Refinement. Refinement of *F*^2^ against ALL reflections. The weighted *R*-factor *wR* and goodness of fit *S* are based on *F*^2^, conventional *R*-factors *R* are based on *F*, with *F* set to zero for negative *F*^2^. The threshold expression of *F*^2^ > σ(*F*^2^) is used only for calculating *R*-factors(gt) *etc*. and is not relevant to the choice of reflections for refinement. *R*-factors based on *F*^2^ are statistically about twice as large as those based on *F*, and *R*- factors based on ALL data will be even larger.

### Fractional atomic coordinates and isotropic or equivalent isotropic displacement parameters (Å^2^)

**Table:**

	*x*	*y*	*z*	*U*_iso_*/*U*_eq_	
Cu1	0.0000	0.5000	1.0000	0.0565 (4)	
N1	0.1018 (4)	0.3629 (5)	0.97413 (19)	0.0449 (13)	
N2	0.0401 (4)	0.4022 (5)	0.85934 (19)	0.0496 (14)	
H2A	0.0758	0.4746	0.8706	0.059*	
H2B	−0.0187	0.4064	0.8767	0.059*	
N3	0.0998 (7)	0.6598 (8)	0.9151 (4)	0.112 (3)	
O1	0.0858 (3)	0.5576 (5)	1.05728 (17)	0.0600 (13)	
O2	0.1714 (4)	0.7103 (5)	1.12935 (19)	0.0683 (14)	
S1	0.1299 (2)	0.8802 (4)	0.84850 (14)	0.1397 (13)	
C1	0.2387 (5)	0.4671 (7)	1.0238 (2)	0.0493 (17)	
C2	0.1830 (5)	0.5544 (7)	1.0579 (2)	0.0484 (16)	
C3	0.2338 (5)	0.6371 (7)	1.0960 (3)	0.0539 (17)	
C4	0.3351 (5)	0.6389 (8)	1.0981 (3)	0.063 (2)	
H4	0.3672	0.6978	1.1224	0.075*	
C5	0.3903 (5)	0.5527 (8)	1.0640 (3)	0.066 (2)	
H5	0.4590	0.5537	1.0657	0.079*	
C6	0.3431 (5)	0.4669 (7)	1.0282 (3)	0.0589 (19)	
H6	0.3800	0.4075	1.0063	0.071*	
C7	0.1925 (5)	0.3692 (7)	0.9874 (2)	0.0525 (17)	
H7	0.2338	0.3034	0.9720	0.063*	
C8	0.0748 (5)	0.2484 (6)	0.9380 (2)	0.0536 (17)	
H8A	0.0056	0.2269	0.9436	0.064*	
H8B	0.1131	0.1690	0.9487	0.064*	
C9	0.0919 (5)	0.2770 (6)	0.8775 (3)	0.0560 (18)	
H9A	0.1622	0.2872	0.8708	0.067*	
H9B	0.0687	0.2002	0.8557	0.067*	
C10	0.0230 (6)	0.4153 (7)	0.7987 (3)	0.064 (2)	
H10	−0.0103	0.3324	0.7857	0.077*	
C11	0.1193 (7)	0.4276 (10)	0.7687 (3)	0.114 (4)	
H11A	0.1546	0.3429	0.7713	0.172*	
H11B	0.1579	0.4991	0.7851	0.172*	
H11C	0.1072	0.4487	0.7304	0.172*	
C12	−0.0447 (7)	0.5355 (8)	0.7880 (3)	0.098 (3)	
H12A	−0.1033	0.5264	0.8101	0.147*	
H12B	−0.0624	0.5378	0.7495	0.147*	
H12C	−0.0112	0.6186	0.7977	0.147*	
C13	0.2134 (6)	0.8058 (8)	1.1669 (3)	0.084 (3)	
H13A	0.2598	0.7600	1.1906	0.127*	
H13B	0.1621	0.8459	1.1890	0.127*	
H13C	0.2468	0.8761	1.1464	0.127*	
C14	0.1133 (7)	0.7473 (10)	0.8862 (4)	0.097 (3)	

### Atomic displacement parameters (Å^2^)

**Table:**

	*U*^11^	*U*^22^	*U*^33^	*U*^12^	*U*^13^	*U*^23^
Cu1	0.0503 (6)	0.0653 (7)	0.0539 (6)	0.0066 (7)	−0.0009 (6)	−0.0144 (7)
N1	0.047 (3)	0.046 (3)	0.041 (3)	−0.004 (3)	0.005 (3)	0.002 (3)
N2	0.058 (3)	0.050 (3)	0.041 (3)	0.013 (3)	0.009 (3)	−0.001 (3)
N3	0.137 (6)	0.072 (5)	0.127 (6)	0.006 (5)	−0.054 (5)	0.004 (5)
O1	0.049 (3)	0.080 (3)	0.051 (3)	0.004 (2)	0.001 (2)	−0.016 (2)
O2	0.069 (3)	0.072 (3)	0.064 (3)	0.003 (3)	−0.011 (3)	−0.018 (3)
S1	0.120 (2)	0.160 (3)	0.139 (3)	0.040 (2)	0.023 (2)	0.056 (2)
C1	0.050 (4)	0.057 (4)	0.041 (3)	0.008 (3)	0.005 (3)	0.007 (3)
C2	0.050 (4)	0.057 (4)	0.039 (4)	−0.001 (3)	−0.004 (3)	0.008 (3)
C3	0.065 (5)	0.051 (4)	0.047 (4)	0.001 (4)	0.001 (4)	0.006 (3)
C4	0.066 (5)	0.063 (5)	0.060 (5)	−0.017 (4)	−0.014 (4)	0.017 (4)
C5	0.050 (5)	0.077 (5)	0.071 (5)	−0.006 (4)	−0.004 (4)	0.015 (4)
C6	0.054 (4)	0.065 (5)	0.058 (4)	0.003 (4)	0.009 (4)	0.013 (4)
C7	0.066 (5)	0.053 (4)	0.039 (4)	0.007 (4)	0.015 (3)	0.004 (3)
C8	0.066 (5)	0.045 (4)	0.050 (4)	0.007 (4)	0.005 (3)	0.001 (3)
C9	0.073 (5)	0.050 (4)	0.046 (4)	0.014 (4)	−0.006 (4)	−0.005 (3)
C10	0.092 (6)	0.061 (5)	0.040 (4)	0.006 (4)	0.001 (4)	−0.001 (4)
C11	0.152 (9)	0.138 (9)	0.053 (5)	0.033 (7)	0.045 (6)	0.019 (5)
C12	0.152 (9)	0.084 (6)	0.058 (5)	0.033 (6)	−0.032 (5)	0.008 (4)
C13	0.109 (7)	0.072 (5)	0.071 (5)	0.003 (5)	−0.020 (5)	−0.019 (4)
C14	0.102 (7)	0.076 (6)	0.114 (8)	0.008 (6)	−0.041 (7)	−0.021 (6)

### Geometric parameters (Å, °)

**Table:**

Cu1—O1^i^	1.891 (4)	C5—C6	1.364 (9)
Cu1—O1	1.891 (4)	C5—H5	0.9300
Cu1—N1^i^	2.023 (5)	C6—H6	0.9300
Cu1—N1	2.023 (5)	C7—H7	0.9300
N1—C7	1.271 (8)	C8—C9	1.504 (8)
N1—C8	1.466 (7)	C8—H8A	0.9700
N2—C9	1.480 (7)	C8—H8B	0.9700
N2—C10	1.486 (7)	C9—H9A	0.9700
N2—H2A	0.9001	C9—H9B	0.9700
N2—H2B	0.9000	C10—C11	1.496 (10)
N3—C14	1.120 (11)	C10—C12	1.513 (9)
O1—C2	1.316 (7)	C10—H10	0.9800
O2—C3	1.370 (7)	C11—H11A	0.9600
O2—C13	1.421 (8)	C11—H11B	0.9600
S1—C14	1.603 (11)	C11—H11C	0.9600
C1—C2	1.404 (8)	C12—H12A	0.9600
C1—C6	1.417 (9)	C12—H12B	0.9600
C1—C7	1.443 (9)	C12—H12C	0.9600
C2—C3	1.405 (9)	C13—H13A	0.9600
C3—C4	1.372 (9)	C13—H13B	0.9600
C4—C5	1.396 (10)	C13—H13C	0.9600
C4—H4	0.9300		
			
O1^i^—Cu1—O1	180	N1—C8—C9	113.3 (5)
O1^i^—Cu1—N1^i^	90.3 (2)	N1—C8—H8A	108.9
O1—Cu1—N1^i^	89.7 (2)	C9—C8—H8A	108.9
O1^i^—Cu1—N1	89.7 (2)	N1—C8—H8B	108.9
O1—Cu1—N1	90.3 (2)	C9—C8—H8B	108.9
N1^i^—Cu1—N1	180.0 (3)	H8A—C8—H8B	107.7
C7—N1—C8	115.3 (6)	N2—C9—C8	111.6 (5)
C7—N1—Cu1	123.2 (5)	N2—C9—H9A	109.3
C8—N1—Cu1	121.4 (4)	C8—C9—H9A	109.3
C9—N2—C10	115.9 (5)	N2—C9—H9B	109.3
C9—N2—H2A	108.1	C8—C9—H9B	109.3
C10—N2—H2A	108.2	H9A—C9—H9B	108.0
C9—N2—H2B	108.6	N2—C10—C11	110.3 (6)
C10—N2—H2B	108.5	N2—C10—C12	109.2 (6)
H2A—N2—H2B	107.4	C11—C10—C12	112.5 (7)
C2—O1—Cu1	127.9 (4)	N2—C10—H10	108.3
C3—O2—C13	118.2 (6)	C11—C10—H10	108.3
C2—C1—C6	119.6 (6)	C12—C10—H10	108.3
C2—C1—C7	121.9 (6)	C10—C11—H11A	109.5
C6—C1—C7	118.4 (6)	C10—C11—H11B	109.5
O1—C2—C1	123.0 (6)	H11A—C11—H11B	109.5
O1—C2—C3	118.8 (6)	C10—C11—H11C	109.5
C1—C2—C3	118.1 (6)	H11A—C11—H11C	109.5
O2—C3—C4	126.0 (7)	H11B—C11—H11C	109.5
O2—C3—C2	112.7 (6)	C10—C12—H12A	109.5
C4—C3—C2	121.3 (7)	C10—C12—H12B	109.5
C3—C4—C5	120.3 (7)	H12A—C12—H12B	109.5
C3—C4—H4	119.8	C10—C12—H12C	109.5
C5—C4—H4	119.8	H12A—C12—H12C	109.5
C6—C5—C4	119.8 (7)	H12B—C12—H12C	109.5
C6—C5—H5	120.1	O2—C13—H13A	109.5
C4—C5—H5	120.1	O2—C13—H13B	109.5
C5—C6—C1	120.8 (7)	H13A—C13—H13B	109.5
C5—C6—H6	119.6	O2—C13—H13C	109.5
C1—C6—H6	119.6	H13A—C13—H13C	109.5
N1—C7—C1	127.2 (6)	H13B—C13—H13C	109.5
N1—C7—H7	116.4	N3—C14—S1	175.6 (11)
C1—C7—H7	116.4		

Symmetry codes: (i) −*x*, −*y*+1, −*z*+2.

### Hydrogen-bond geometry (Å, °)

**Table:**

*D*—H···*A*	*D*—H	H···*A*	*D*···*A*	*D*—H···*A*
N2—H2A···N3	0.90	2.13	2.972 (10)	155
N2—H2B···O1^i^	0.90	1.87	2.665 (6)	147

Symmetry codes: (i) −*x*, −*y*+1, −*z*+2.
